# Genomics of Tumor Origin and Characteristics for Adenocarcinoma and Malignant Pleural Mesothelioma: A Case Report

**DOI:** 10.3389/fonc.2022.858094

**Published:** 2022-05-19

**Authors:** Katsuo Usuda, Yo Niida, Masahito Ishikawa, Shun Iwai, Aika Yamagata, Yoshihito Iijima, Nozomu Motono, Sohsuke Yamada, Hidetaka Uramoto

**Affiliations:** ^1^ Department of Thoracic Surgery, Kanazawa Medical University, Kahoku-gun, Japan; ^2^ Department of Rehabilitation Medicine, Shimada Hospital, Fukui, Japan; ^3^ Center for Clinical Genomics, Kanazawa Medical University, Kahoku-gun, Japan; ^4^ Division of Genomic Medicine, Kanazawa Medical University, Kahoku-gun, Japan; ^5^ Department of Pathology and Laboratory Medicine, Kanazawa Medical University, Kahoku-gun, Japan

**Keywords:** lung cancer, genomics, oncogene, adenocarcinoma, malignant pleural mesothelioma

## Abstract

A female underwent a right middle lobectomy for a pulmonary adenocarcinoma (AD). She eventually died of a right malignant pleural mesothelioma (MPM; sarcomatoid type) 4 years and 7 months after the removal of the AD even though she did not have any history of asbestos exposure, smoking, or radiation exposure. Her chest CT revealed multiple pulmonary nodules and bilateral pleural effusion with a right pleural tumor directly invading into the abdominal cavity. The genomics of tumor origin and characteristics were examined for the AD and the MPM. As a result, 50 somatic variants were detected in the AD, and 29 somatic variants were detected in the MPM. The variants which were common in both the AD and the MPM were not present, which suggested that the AD and the MPM had occurred independently in different origins. The MPM had two driver oncogenes of *TP53* and *EP300*, but the AD did not. Two driver oncogenes of *TP53* and *EP300* were hypothesized to make the MPM aggressive. The speed at which the MPM progressed without the patient having a history of asbestos exposure, smoking, or radiation exposure was alarming.

## Introduction

Malignant mesothelioma (MM) is an aggressive malignancy of serosal membranes, including the pleura, peritoneum, pericardium, and the tunica vaginalis of the testes, predominantly caused by prior asbestos exposure (1). Malignant pleural mesothelioma (MPM) is the most common form of mesothelioma, accounting for approximately 80% of the disease, and is a lethal cancer with nearly 25,000 deaths worldwide in 2018 (2). Despite global efforts to reduce asbestos exposure through prohibition and mine closure in many countries, a decrease of mesothelioma incidences has not been achieved. It has been characterized by a long latency period between asbestos exposure and MPM presentation (13–70 years) and a lower survival rate (3). Some studies average the prognosis to be roughly 1 year after diagnosis (4). Genetic changes are required for the malignant transformation of mesothelial cells. Several oncogenes and tumor suppressors have been hypothesized to play a role in MPM carcinogenesis (5). MPM is histopathologically classified into three variants: epithelioid, sarcomatoid, and mixed/biphasic (6).

We experienced an impressive case in whom an aggressive MPM was diagnosed 4 years and 7 months after a curative pulmonary resection for pulmonary adenocarcinoma (AD). The origin and characteristics for the two kinds of tumors were examined from the point of genomics.

## Background

**Figure 1:**
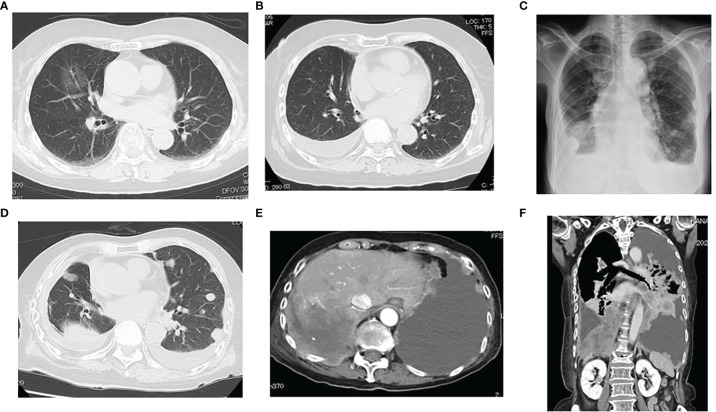
**(A)** Chest CT showing a ground glass opacity of 32 mm in size in her middle lobe of the right lung. **(B)** At 2 years and 3 months after pulmonary resection, the follow-up chest CT revealed right pleural effusion. **(C–F)** At 4 year and 7 months after pulmonary resection, the chest CT revealed multiple pulmonary nodules and bilateral pleural effusion with a right pleural tumor directly invading into the abdominal cavity. A right malignant pleural mesothelioma (sarcomatoid type) invaded to the right adrenal gland and the liver.

**Figure 2:**
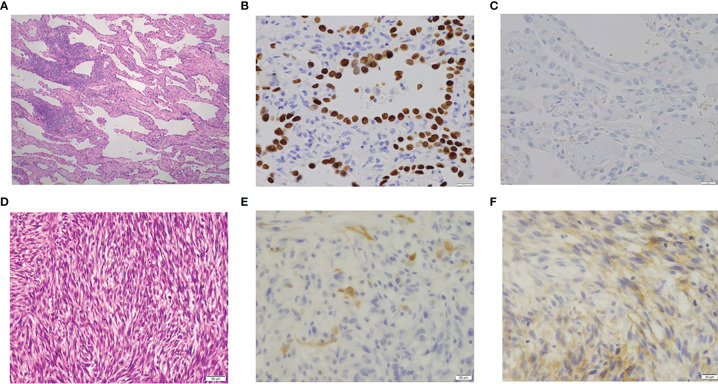
**(A)** Pathology of primary lung cancer (×100). **(B)** TTF-1 of lung cancer (positive; ×400). **(C)** Calretinin of lung cancer (negative; ×400). **(D)** Pathology of malignant pleural mesothelioma (×200). **(E)** Calretinin of malignant pleural mesothelioma (MPM; weakly positive; ×400). **(F)** D2-40 of MPM (positive; ×400).

A 77-year-old female patient did not have a history of asbestos exposure, smoking, or radiation exposure. She used to live in the country and was a housewife. She did not work in a factory and had no known contact with asbestos. She underwent a right middle lobectomy and nodal dissection for pulmonary AD which showed a ground glass opacity of 32 mm in size (**Figure 1A**). The diagnosis of AD was determined to be a minimally invasive AD (predominantly lepidic pattern) of pT2aN0M0 (pStage IB). For this diagnosis, we used not only morphological methods but also immunohistochemistry methods (**Figures 2A–C**). The AD was positive for TTF-1 and Napsin A and negative for calretinin, D2-40, or p40, which meant a pulmonary origin. Its epidermal growth factor receptor in real-time PCR was negative, and its anaplastic lymphoma kinase was negative. After the pulmonary resection, the patient had follow-up chest X-ray or chest CT every 6 months. At 2 years and 3 months after the pulmonary resection, the follow-up chest CT revealed a right pleural effusion (**Figure 1B**). The cytology of the right pleural effusion was negative for malignancy, and a recurrence of lung cancer was judged to be negative. At 4 years and 5 months after the pulmonary resection, there was not any symptom and any evidence of another tumor. At 4 years and 7 months after the pulmonary resection, the chest CT revealed multiple pulmonary nodules and bilateral pleural effusion with a right pleural tumor directly invading into the abdominal cavity (**Figures 1C–F**). The brain CT revealed a brain metastasis. The cytology of the right pleural effusion gave two negative results. The patient died of respiratory failure due to the malignant tumors within a month after the chest CT. An autopsy of the patient revealed that a right MPM (sarcomatoid type) invaded into the right adrenal gland and the liver with multiple pulmonary metastasis, multiple pleural metastasis, multiple metastasis to hilar and mediastinal lymph nodes, and oligometastasis into the heart. The MPM consisted of fusiform-shaped sarcomatoid mesothelial cells. The MPM was negative for TTF-1, Napsin A, or p40 and weakly positive for calretinin and positive for D2-40 (**Figures 2D–F**), which meant a mesothelium origin. The pathology was quite different from the adenocarcinoma.

**Figure 3:**
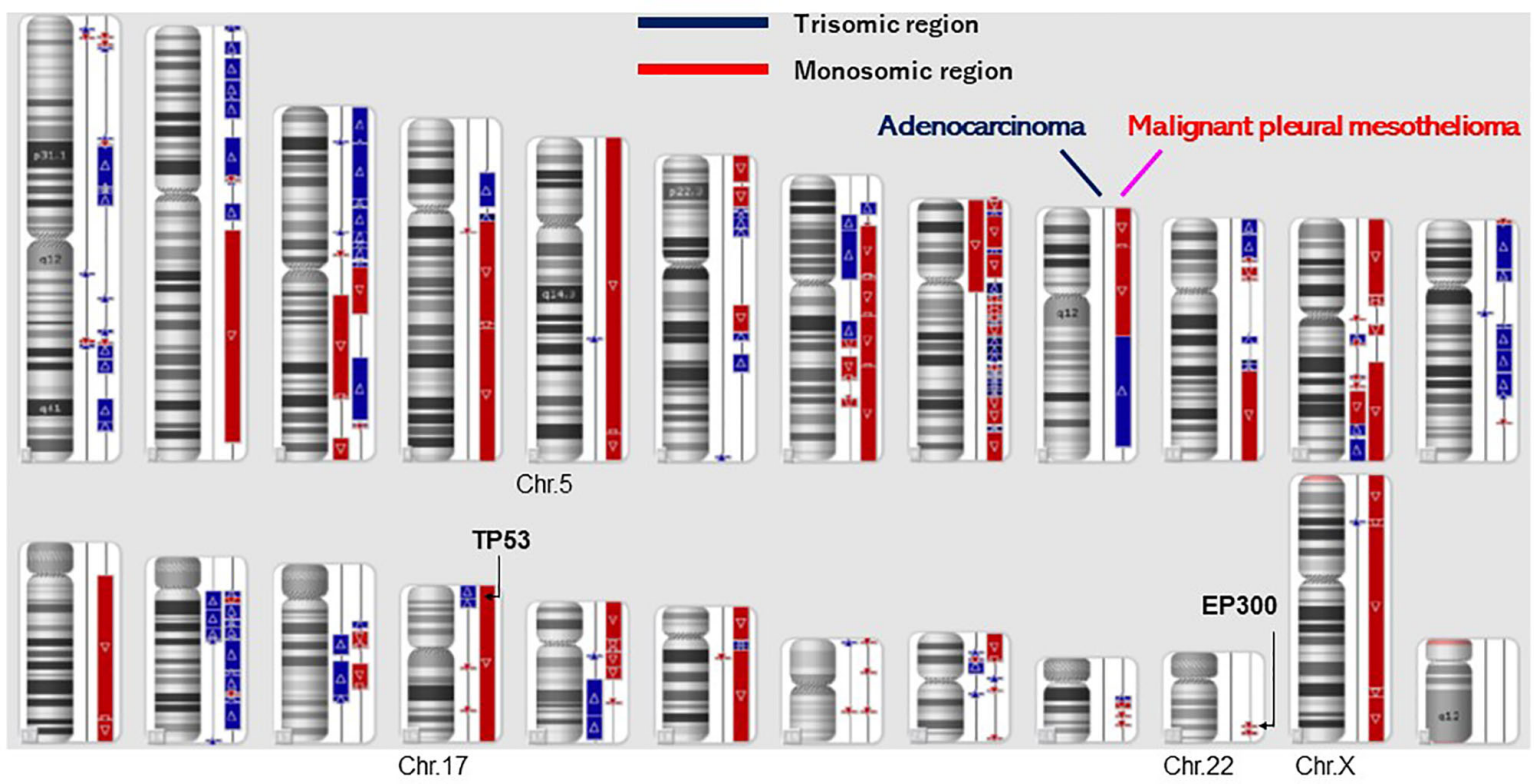
Structural chromosomal aberration analysis by DNA microarray (OncoScan CNV). All chromosomes showed abnormality in malignant pleural mesothelioma, whereas the chromosomal aberration of lung adenocarcinoma was relatively local. A commonality was not found in the patterns of the chromosomal aberration between the adenocarcinoma and the malignant pleural mesothelioma.

A dual deep sequence was performed to accurately compare the genomes of the two tumors by SureSelect NCC oncopanel (Agilent) (7). Two kinds of DNA polymerase (KAPA, Roche CustomBiotech; NEB, New England Biolabs) were employed for the first (pre-capture) library amplification, then two libraries were created for each of the AD and the MPM. Variant calls common to the KAPA and NEB libraries were selected in each tumor, and variant calls with variant allele frequency (VAF) less than 5% (<0.05) were eliminated as noise (7). As a result, 50 somatic variants were detected in the AD, and 29 somatic variants were detected in the MPM. The variants which were common both in the AD and the MPM were not present, which suggested that the origins of these tumors were different. The tumor content ratio was low (25 to 30%) in the adenocarcinoma, and all the detected mutations seemed to be heterozygous mutations (**Table 1**). Mutations of *TP53* and *EP300* were detected in MPM, not in AD. Although a specific driver mutation was not detected in AD, the mutations of *PIK3R1* c.1915C>T p.(Arg639Ter) and *FLT3* c.931C>T p.(Arg311Trp) were there, which were registered in the Catalogue of Somatic Mutations in Cancer (COSIMC). The tumor mutation burden (TMB) was 53.0 mut/Mbp in the AD and 30.7 mut/Mbp in the MPM, whose TMBs showed a higher hypermutation rate. There was no translocation of chromosome and no detection of fused genes in the AD and the MPM. As for the mutation of *TP53* and *EP300*, VAF was high in MPM, the tumor content ratio was about 90%, and the mutations were interpreted as with accompanying homozygosity and loss of heterozygosity (LOH). The two tumors had different molecular pathologies and different origins. In the structural chromosomal aberration analysis by DNA microarray with OncoScan CNV (Affymetrix) (**Figure 3**), all chromosomes of the MPM showed abnormality, whereas the chromosomal aberration of AD showed relatively local abnormality. The commonality was not found in the pattern of the chromosomal aberration between the AD and the MPM. The entire chromosome 17 and *EP300* locus of chromosome 22 of MPM exhibited a single copy, and LOH of *TP53* and *EP300* was confirmed to be due to chromosome deletion.

**Table 1:** Suspicious pathogenic variants detected by cancer gene panel.

Tumor	Chromosome number	POS(hg38)	dbSNP_ID	REF	ALT	Gene	HGVS_Format	TV_GT	VAF	SIFT	PP2HVAR	PP2HDIV	MUTTASTER	MUTASSESSOR	LRT
AD	chr9	136502399	–	C	T	*NOTCH1*	NM_017617.5:c.5257G>A p.(Gly1753Arg)	Hetero	0.16	D(0.034)	P(0.9)	D(0.999)	D(1)	M(2.74)	D(0.000001)
	chr5	56881876	–	C	A	*MAP3K1*	NM_005921:c.2676C>A p.(Asn892Lys)	Hetero	0.14	D(0.008)	B(0.006)	B(0.006)	N(0.894783)	L(1.7)	N(0.052341)
	chr13	28049489	–	G	A	***FLT3***	NM_004119.3:c.931C>T p.(Arg311Trp)	Hetero	0.13	D(0.002)	D(0.999)	D(1.0)	D(0.999705)	L(0.805)	D(0.000004)
	chr5	68296271	–	C	T	***PIK3R1***	NM_001242466.2:c.1915C>T p.(Arg639Ter)	Hetero	0.12						
	chr3	47037710	–	C	A	*SETD2*	NM_001349370.3:c.7306G>T p.(Glu2436Ter)	Hetero	0.1						
	chr19	17840301	–	G	T	*JAK3*	NM_000215.4:c.1183C>A p.(Arg395Ser)	Hetero	0.07	T(0.063)	B(0.042)	B(0.046)	N(1)	N(0)	N(0.846991)
	chr19	10499591	–	C	A	*KEAP1*	NM_012289.4:c.443G>T p.(Gly148Val)	Hetero	0.06	D(0.039)	D(0.974)	D(0.999)	D,D(1,1)	L(1.67)	D(0.000000)
	chr9	132910576	rs118203506	TG	TGG	*TSC1*	NM_000368.5:c.1256dupC p.(Arg420fs)	Hetero	0.06						
	chr16	346821	–	C	A	*AXIN1*	NM_003502.4:c.205G>T p.(Gly69Trp)	Hetero	0.05	D(0.001)	D(1.0)	D(1.0)	D,D(1,1)	M(2.54)	D(0.000000)
MPM	chr17	7675124	rs148924904	T	C	***TP53***	NM_000546.6:c.371A>G p.(Tyr124Cys)	Homo	**0.89**	D(0.0,0.0)	D(0.999)	D(1.0)	D(0.999992)	M(2.14)	D(0.000003)
	chr22	41117297	–	G	C	***EP300***	NM_001362843.2:c.205G>C p.(Gly69Arg)	Homo	**0.87**	D(0.002)	D(0.971)	D(0.999)	D(0.991824)	L(1.65)	D(0.000141)
	chr9	136505577	rs778742968	A	G	*NOTCH1*	NM_017617.5:c.4319T>C p.(Ile1440Thr)	Hetero	0.1	T(0.264)	D(0.996)	D(0.999)	D(0.99977)	N(-0.14)	U(0.000000)
	chr1	64855540	–	C	A	*JAK1*	NM_001320923.2:c.1617G>T p.(Met539Ile)	Hetero	0.09	T(0.136)	B(0.0)	B(0.0)	N(0.999428)	N(0)	
	chr19	15191656	–	GCCTGTGGCACACAGATGCAGCAGTCCAGCCACCTGGCGCATGTCCACCCGAGGCCTGCCTCCCCGCTCCCTCTGGCCGCAGTGCCCA	G	*NOTCH3*	NM_000435.3:c.802+2_803del p.(Gly268fs)	Hetero	0.09						
	chr9	95508310	rs756897237	TGCC	T	*PTCH1*	NM_000264.5:c.49_51del p.(Gly17del)	Hetero	0.08						
	chr7	129206365	–	CGCAGGTATAGTGACTGGTAGGAACGGGAGACCTGGATGGGGTGAGTTTGAGGGAGGGGGCCAGTAACCCACCTTCTGTCCCACCCCTTCCTGCT	C	*SMO*	NM_005631.5:c.1140+3_1142del p.(Val381fs)	Hetero	0.06						
	chr1	11139434	–	GCCTTAAAAATAAGAGAAACTGGGTTATAGACAGAACTGGACAGCCCAGGGACACCATGGGGCCCTACCTGCCCATGTGGGTGGGTGGTTGTCACTCA	G	*MTOR*	NM_001386500.1:c.4998+2_4999del p.(Ala1667fs)	Hetero	0.06						
	chr9	136504956	rs761020817	GCAC	G	*NOTCH1*	NM_017617.5:c.4732_4734del p.(Val1578del)	Hetero	0.06						
	chr9	136515399	–	TCCTGAAGGGGTGGCACGTGTCGGTCAGTCCTCAGGCCCGCCCTGCCCACTGGCCCCCCGCCGGCCACCCGCCTGGCCGGCCA	T	*NOTCH1*	NM_017617.5:c.1903+2_1904del p.Gly635fs	Hetero	0.06						
	chr4	54274562	–	AGTCCTGGTGCTGTTGGTGATTGTGATCATCTCACTTATT	A	*PDGFRA*	NM_001347827.2:c.1599_1637del p.(Leu534_Val546del)	Hetero	0.06						

AD, adenocarcinoma; MPM, malignant pleural mesothelioma; TV_GT, zygosity of the mutations in tumors estimated from tumor cell content and VAF; VAF, variant allele frequency; in silico analysis was performed by Variant Annotation Integrator (https://genome.ucsc.edu/cgi-bin/hgVai); SIFT, sorting intolerant from tolerant (D, damaging; T, tolerated); PP2HVAR, PolyPhen-2 with HumVar training set (D, probably damaging; P, possibly damaging; B, benign); PP2HVAR, PolyPhen-2 with HumDiv training set (D, probably damaging; P, possibly damaging; B, benign); MUTTASTER, MutationTaster (D, disease causing; N, polymorphism); MUTASSESSOR, Mutationassessor (M, medium; L, low; N, neutral); LRT, likelihood ratio test (D, deleterious; N, Neutral; U, unknown).

## Discussion

The patient did not have any history of asbestos exposure, smoking, occupational radiation exposure, or medical radiation therapy. Besides these, there was no evidence of mesothelioma in the right pleural cavity when she underwent a right middle lobectomy and nodal dissection for pulmonary AD. Although we should have had performed more detailed examinations to detect the new tumor at 4 years and 5 months after the pulmonary resection, we did not do so because we did not suspect the recurrence of the adenocarcinoma nor a newly developed tumor. The MPM was an extremely aggressive malignant tumor that progressed quickly, causing the patient to die within 2 months.

We evaluated the patient and analyzed the causes and the genomics of the AD and the MPM. The pulmonary AD was a minimally invasive AD (predominantly lepidic pattern) of pT2aN0M0 (pStage IB) and was removed by resection. In the literature, there were several case reports in which malignant pleural mesotheliomas invaded into the lung parenchyma with intrapulmonary lepidic spread (8, 9). In our case, the immunohistochemistry indicated that the adenocarcinoma originated from the right lung and was a typical minimally invasive AD with a predominantly lepidic pattern. Besides this, the immunohistochemistry indicated that the MPM originated from the mesothelium. Our case is quite different from such cases of a malignant pleural mesothelioma with intrapulmonary lepidic spread. An autopsy of the patient was performed to assess the cause of death. The patient died from the aggressive mesothelioma. The patient’s case was discussed by surgeons and pathologists. This research began postmortem, so we were not able to get her perspective.

Although asbestos was certainly the largest and most well-known cause of MM, roughly 20% of the patients did not have any known exposure to asbestos (4). A statistically significant increase of MM develops following radiation therapy for breast cancer, testicular cancer, Hodgkin’s lymphoma, and non-Hodgkin’s lymphoma (10–13). A couple of causal factors for MPM include occupational radiation exposure and medical radiation therapy (14). Our patient had no history of exposure to either of these causal factors. The rate of sarcomatoid and biphasic disease is higher in the pleura compared to the peritoneum. A pleural MM occurs much more commonly in men, while a peritoneal MM occurs in younger patients. Pleural MM has a relatively lower 5-year survival, even for the more favorable epithelioid histology. In contrast, it has been observed that a subset of epithelioid peritoneal MM patients had prolonged survival following an aggressive therapy. Collectively, these observations raise the possibility that pleural MM and peritoneal MM have a similar morphological representation but are biologically distinct from each other.

We found the main driver mutations of *TP53* (p53) and *EP300* in the MPM and hypothesized that these driver mutations made the MPM aggressive. *TP53* was reported in Li-Fraumeni syndrome, which was an autosomal dominant inheritance disease of pathologic mutation of the *TP53* gene in the germline on ClinVar. *TP53* is the most frequently mutated gene (>50%) in human cancer, indicating that the *TP53* gene plays a crucial role in preventing cancer formation. The *TP53* gene is located on the short arm of chromosome 17 (17p13.1). MPMs with *TP53* mutations were reported to have a more aggressive phenotype (15). The mutation of the *TP53* gene predicted shorter survival (16). A univariate regression analysis revealed that the overexpression of *TP53* and B-cell lymphoma-2-associated X protein (BAX) in colorectal cancer tissues was associated with poor patient outcome (17). *TP53* is associated with important cell functions, such as the termination on the border, apoptosis instruction, DNA repair promotion, and neovascularization suppression. *EP300*, also known as histone acetyltransferase p300, E1A-associated protein p300, or p300, is an enzyme that is encoded by the *EP300* gene. *EP300* mutations contribute to an unfavorable phenotype in a number of solid tumors and hematological malignancies, and therefore *EP300* is often considered as a tumor suppressor (18). This enzyme plays an essential role in regulating cell growth and division, prompting cells to mature and preventing the growth of cancerous tumors. The downregulation of *EP300* gene expression was associated with higher anti-tumor immunity in most solid malignancies (19). The *EP300* gene is located on the long arm of chromosome 22 (22p13.2). *EP300* and *BAX* contribute to the regulation of the cell cycle and apoptosis, cellular processes that are often impaired in cancer cells. Dysregulations of the expression of *EP300*, *TP53*, and *BAX* genes were found to contribute to colorectal cancer pathogenesis (17).

MPM is characterized by a low mutation load (20). MPM does not appear to be involved in the aberrant expression of many well-studied growth control genes, such as *HRAS*, *KRAS*, *TP53* (p53), and *RB1* (21–23), although the SV40 T antigen has been proposed to inactivate p53 function in some MPM tumors (24). MPM was reported to be characterized by the frequent inactivation of tumor suppressor genes, *e*.*g*., the homozygous deletion of the cyclin-dependent kinase inhibitor 2A/2B, various genetic alterations that inactivate BRCA1-associated protein-1 (*BAP1*), neurofibromin 2, large tumor-suppressor kinase 2, and tumor protein p53 (*TP53*) (4, 25–27). In our case study, the mesothelioma did not have either the BAP1 or CDKN2A mutation. The impact that the BAP1 and CDKN2A mutations have on MPM is unclear. *TP53* and *RB1* tumor suppressor genes were important in maintaining genetic homeostasis in MPM (28).

Although a specific driver mutation was not detected in the adenocarcinoma, there were mutations with registration in COSIMC. *PIK3R1* c.1915C>T p.(Arg639Ter) is a mutation with registration in COSIMC, and pathologic significance is confirmed (COSV57126125). The mutation was observed in colorectal cancers and prostate cancers (https://cancer.sanger.ac.uk/cosmic/search?q=PIK3R1+c.1915C). FLT3 c.931C>T p.(Arg311Trp) is also a mutation with registration in COSIMC, and pathologic significance is confirmed (COSV54057282). The mutation was observed in colorectal cancers and prostate cancers (https://cancer.sanger.ac.uk/cosmic/search?q=FLT3+c.931C). As a result, *PIK3R1* and *FLT3* were recognized to be mutations that were not correlated to metastasis and the recurrence of a malignant tumor.

The patterns of chromosomal aberration of the AD and the MPM were quite different. The AD and the MPM showed different patterns in the chromosome structure analysis by OncoScan; specifically, the MPM had structural abnormalities in all chromosomes. The NCC Oncopanel showed no pathologic fusion gene in the AD and the MPM. The two kinds of tumor had different molecular pathologies and occurred in different origins. The TMB was 53.0 mut/Mbp in the AD and 30.7 mut/Mbp in the MPM. The TMBs showed higher hypermutation rates, and immune checkpoint inhibitors could be effective for patient therapy.

## Concluding Remarks

AD and MPM occurred independently and had different origins. The MPM had the two oncogenes of *TP53* and *EP300*, but the AD did not. The two driver oncogenes of *TP53* and *EP300* were hypothesized to make the MPM more aggressive. The MPM progressed quickly without a history of asbestos exposure, smoking, or radiation exposure.

## Data Availability Statement

The original contributions presented in the study are included in the article/supplementary material. Further inquiries can be directed to the corresponding author.

## Ethics Statement

Ethical review and approval were not required for the study on human participants in accordance with the local legislation and institutional requirements. The patients/participants provided their written informed consent to participate in this study. Written informed consent was obtained from the individual(s) for the publication of any potentially identifiable images or data included in this article.

## Author Contributions

KU performed the research and wrote the paper. MI, SI, AY, YI, and NM performed therapy on a patient. YN contributed to the analysis of the genetic status of this patient. SY performed a pathological examination of lung cancers. HU contributed to the supervision of this study and revision of the manuscript. Dustin Keeling, whose native language is English, revised the paper. All authors contributed to the article and approved the submitted version.

## Funding

This research was supported partly by a grant-in-aid for scientific research from the Ministry of Education, Culture, Sports, Science and Technology, Japan (grant number: 20K09172).

## Conflict of Interest

The authors declare that the research was conducted in the absence of any commercial or financial relationships that could be construed as a potential conflict of interest.

## Publisher’s Note

All claims expressed in this article are solely those of the authors and do not necessarily represent those of their affiliated organizations, or those of the publisher, the editors and the reviewers. Any product that may be evaluated in this article, or claim that may be made by its manufacturer, is not guaranteed or endorsed by the publisher.

## References

[1] Robinson BM. Malignant Pleural Mesothelioma: An Epidemiological Perspective. Ann Cardiothorac Surg (2012) 1:491–6. doi: 10.3978/j.issn.2225-319X.2012.11.04 PMC3741803 23977542

[2] Bray F Ferlay J Soerjomataram I Siegel RL Torre LA Jemal A. Global Cancer Statistics 2018: GLOBOCAN Estimates of Incidence and Mortality Worldwide for 36 Cancers in 185 Countries. CA Cancer J Clin (2018) 68:394–424. doi: 10.3322/caac.21492 30207593

[3] Lanphear BP Buncher CR. Latent Period for Malignant Mesothelioma of Occupational Origin. J Occup Med (1992) 34:718–21. 1494965

[4] Abbott DM Bortolotto C Benvenuti S Lancia A Filippi AR Stella GM. Malignant Pleural Mesothelioma: Genetic and Microenviromental Heterogeneity as an Unexpected Reading Frame and Therapeutic Challenge. Cancers (2020) 12(5):1186. doi: 10.3390/cancers12051186 PMC7281319 32392897

[5] Lehman TA Reddel R Peiifer AM Spillare E Kaighn ME Weston A. Oncogenes and Tumor-Suppressor Genes. Environ Health Perspect (1991) 93:133–4. doi: 10.1289/ehp.9193133 PMC1568035 1685442

[6] Galateau-Salle F Churg A Roggli V Travis WD. World Health Organization Committee for Tumors of the Pleura. The 2015 World Health Organization Classification of Tumors of the Pleura: Advances Since the 2004 Classification. J Thorac Oncol (2016) 11:142–54. doi: 10.1016/j.jtho.2015.11.005 26811225

[7] Hiroki U Sumihito T Yo N. Dual Deep Sequencing Improves the Accuracy of Low-Frequency Somatic Mutation Detection in Cancer Gene Panel Testing. Int J Mol Sci (2020) 21:3530. doi: 10.3390/ijms21103530 PMC7278996 32429412

[8] Rossi G Cavazza A Turrini E Costantini M Casali C Morandi. Exclusive Intrapulmonary Lepidic Growth of a Malignant Pleural Mesothelioma Presenting With Pneumothorax and Involving the Peritoneum. Int J Surg Pathol (2006) 14(3):234–7. doi: 10.1177/1066896906290360 16959711

[9] Jimbo N Kawahara K Tsukamoto Minami K Tanaka Y Maniwa Y. Malignant Pleural Mesothelioma Showing Intrapulmonary Lepidic Spread. Pathol Int (2020) 70(6):373–5. doi: 10.1111/pin.12927 32243039

[10] Tward JD Wendland MM Shrieve DC Szabo A Gaffney DK. The Risk of Secondary Malignancies Over 30 Years After the Treatment of Non-Hodgkin Lymphoma. Cancer (2006) 107:108–15. doi: 10.1002/cncr.21971 16708354

[11] Teta MJ Lau E Sceurman BK Wagner ME. Therapeutic Radiation for Lymphoma: Risk of Malignant Mesothelioma. Cancer (2007) 109:1432–8. doi: 10.1002/cncr.22526 17315168

[12] Travis LB Fossa SD Schonfeld SJ McMaster ML Lynch CF Storm H. Second Cancers Among 40,576 Testicular Cancer Patients: Focus on Long-Term Survivors. J Natl Cancer Inst (2005) 97:1354–65. doi: 10.1093/jnci/dji278 16174857

[13] Brown LM Chen BE Pfeiffer RM Schairer C Hall P Storm H. Risk of Second Non-Hematological Malignancies Among 376,825 Breast Cancer Survivors. Breast Cancer Res Treat (2007) 106:439–51. doi: 10.1007/s10549-007-9509-8 17277968

[14] Cavazza A Travis LB Travis WD Wolfe JT Foo ML Gillespie DJ. Post-Irradiation Malignant Mesothelioma. Cancer (1996) 77:1379–85. doi: 10.1002/(SICI)1097-0142(19960401)77:7<1379::AID-CNCR24>3.0.CO;2-Y 8608519

[15] Lo Iacono M Monica V Righi L Grosso F Libener R Vatrano S. Targeted Next-Generation Sequencing of Cancer Genes in Advanced Stage Malignant Pleural Mesothelioma: A Retrospective Study. J Thorac Oncol (2015) 10:492–9. doi: 10.1097/JTO.0000000000000436 25514803

[16] May P May E. Twenty Years of P53 Research: Structural and Functional Aspects of the P53 Protein. Oncogene (1999) 18:7621–36. doi: 10.1038/sj.onc.1203285 10618702

[17] Kowalczyk AE Krazinski BE Godlewski J Kiewisz J Kwiatkowski P Sliwinska-Jewsiewicka A. Expression of the EP300, TP53 and BAX Genes in Colorectal Cancer: Correlations With Clinicopathological Parameters and Survival. Oncol Rep (2017) 38:201–10. doi: 10.3892/or.2017.5687 28586030

[18] Godlewski J Krazinski BE Kowalczyk AE Kiewisz J Kiezun J Kwiatkowski P. Expression and Prognostic Significance of EP300, TP53 and BAX in Clear Cell Renal Cell Carcinoma. Anticancer Res (2017) 37:2927–37. doi: 10.21873/anticanres.11646 28551630

[19] Krupar R Watermann C Idel C Ribbat-Idel J Offermann A Pasternack H. *In Silico* Analysis Reveals EP300 as a Pancancer Inhibitor of Anti-Tumor Immune Response *via* Metabolic Modulation. Sci Rep (2020) 10:9389. doi: 10.1038/s41598-020-66329-7 32523042 PMC7287052

[20] Chalmers ZR Connelly CF Fabrizio D Gay L Ali SM Ennis R. Analysis of 100,000 Human Cancer Genomes Reveals the Landscape of Tumor Mutational Burden. Genome Med (2017) 9(1):34. doi: 10.1186/s13073-017-0424-2 28420421 PMC5395719

[21] Metcalf RA Welsh JA Bennet WP Seddon MB Lehman TA Pelin K. P53 and Kirsten-Ras Mutations in Human Mesothelioma Cell Lines. Cancer Res (1992) 52:2610 –5. 1568228

[22] Mor O Yaron P Huszar M Yellin A Kakobovitz O Brok-Simoni F. Absence of P53 Mutations in Malignant Mesotheliomas. Am J Respir Cell Mol Biol (1997) 16:9 –13. doi: 10.1165/ajrcmb.16.1.8998073 8998073

[23] Gavin J Gordon Graham N Rockwell Roderick V Jensen. Identification of Novel Candidate Oncogenes and Tumor Suppressors in Malignant Pleural Mesothelioma Using Large-Scale Transcriptional Profiling. Am J Pathol (2005) 166:1827–40. doi: 10.1016/S0002-9440(10)62492-3 PMC1363736 15920167

[24] Carbone M Rizzo P Grimley PM Procopio A Mew DJY Shridhar V. Simian Virus-40 Large-T Antigen Binds P53 in Human Mesothelioma. Nat Med (1997) 3:908 –12. doi: 10.1038/nm0897-908 9256284

[25] Yang H Xu D RA S Peng RW. Biomarker-Guided Targeted and Immunotherapies in Malignant Pleural Mesothelioma. Ther Adv Med Oncol (2020) 12:1758835920971421. doi: 10.1177/1758835920971421 33240401 PMC7672749

[26] Bott M Brevet M Taylor BS Shimizu S Ito T Wang L. The Nucleardeubiquitinase BAP1 Is Commonly Inactivated by Somatic Mutations and 3p21.1 Losses in Malignant Pleural Mesothelioma. Nat Genet (2011) 43(7):668–72. doi: 10.1038/ng.855 PMC4643098 21642991

[27] Cokiroglu E Senturk S. Genomics and Functional Genomics of Malignant Pleural Mesothelioma. Int J Mol Sci (2020) 21(17):6342. doi: 10.3390/ijms21176342 PMC7504302 32882916

[28] Røe OD Stella GM. Malignant Pleural Mesothelioma: History, Controversy and Future of a Manmade Epidemic. Eur Respir Rev (2015) 24:115–31. doi: 10.1183/09059180.00007014 PMC9487774 25726562

